# The prevalence and possible risk factors of gaming disorder among adolescents in China

**DOI:** 10.1186/s12888-024-05826-9

**Published:** 2024-05-21

**Authors:** Lina Zhang, Jiaqi Han, Mengqi Liu, Cheng Yang, Yanhui Liao

**Affiliations:** 1 Department of Clinical Psychology, Tianjin Medical University General Hospital, Tianjin, China; 2 The Clinical Medical of Xinjiang Medical University, Urumqi, China; 3 The National Clinical Research Center for Mental Disorders and Beijing Key Laboratory of Mental Disorders and Beijing Institute for Brain Disorders Center of Schizophrenia, Beijing Anding Hospital, Capital Medical University, Beijing, China; 4 Advanced Innovation Center for Human Brain Protection, Capital Medical University, Beijing, China; 5 National Clinical Research Center for Mental Disorders, Department of Psychiatry, The Second Xiangya Hospital of Central South University, Changsha, China; 6 Department of Psychiatry, Sir Run Run Shaw Hospital, School of Medicine, Zhejiang University, Hangzhou, China

**Keywords:** Gaming disorder, Prevalence, Possible risk factors, Adolescents, China

## Abstract

**Background:**

Nowadays, moderate gaming behaviors can be a pleasant and relaxing experiences among adolescents. However, excessive gaming behavior may lead to gaming disorder (GD) that disruption of normal daily life. Understanding the possible risk factors of this emerging problem would help to suggest effective at preventing and intervening. This study aimed to investigate the prevalence of GD and analyze its possible risk factors that adolescents with GD.

**Methods:**

Data were collected between October 2020 and January 2021. In total, a sample of 7901 students (4080 (52%) boys, 3742 (48%) girls; aged 12–18 years) completed questionnaires regarding the Gaming-Related Behaviors Survey, Gaming Disorder Symptom Questionnaire-21 (GDSQ-21); Behavioral Inhibition System and Behavioral Activation System Scale (BIS/BAS Scale); Emotion Regulation Questionnaire (ERQ); Short-form Egna Minnenav Barndoms Uppfostran for Chinese (s-EMBU-C); and Adolescent Self-Rating Life Events Checklist (ASLEC).

**Results:**

The prevalence of GD was 2.27% in this adolescent sample. The GD gamers were a little bit older (i.e., a higher proportion of senior grades), more boys, with more gaming hours per week in the last 12 months, with more reward responsiveness, maternal rejecting and occurrence of negative life events (e.g., interpersonal relationships, being punished and bereavement factors).

**Conclusion:**

These possible risk factors may influence the onset of GD. Future research in clinical, public health, education and other fields should focus on these aspects for provide target prevention and early intervention strategies.

**Supplementary Information:**

The online version contains supplementary material available at 10.1186/s12888-024-05826-9.

## Introduction

Video games (typically online games) have become an immensely popular form of entertainment for adolescents. Although moderate gaming has been found to be associated with relaxation and stress reduction [1–3], excessive use of games may take an uncontrolled addictive form in some individuals [4–8]. Psychiatrists and other health professionals have suggested that excessive use of gaming may cause negative outcomes for interpersonal relationships, physical well-being, mental health, education, and employment [9]. In 2013, the Fifth Edition of Diagnostic and Statistical Manual of Mental Disorders (DSM-5) introduced Internet gaming disorder (henceforth referred to as IGD) and suggested it as a possible condition for further study [10]. In June 2018, the 11th Revision of the International Classification of Diseases (ICD-11) included Gaming Disorder (henceforth referred to as GD) as a disease entity. GD is characterized by impaired control over gaming, increasing prioritization of gaming over other life interests and daily activities, and continuation or escalation of gaming despite negative consequences [11]. For adolescents, the prevalence of IGD has been reported to be 9.9% (CI = 1.0-21.5%) in Asia, 9.4% (95% CI = 8.3-10.5%) in North America, 4.4% (95% CI = 1.9-7.4%) in Australia and 3.9% (95% CI = 2.8-5.3%) in Europe [12]. In addition, the present research shows that individuals’ gaming behaviours cause not only functional impairments in main areas but also other related mental health issues. Thus, preventing GD seems to be of essential importance, as shown by calls for preventive interventions for groups at high risk of GD (e.g., adolescents) at the social, family and school levels [13]. To prevent GD from developing, we need to investigate the China prevalence of GD and analyze the possible risk factors that problematic gaming behaviour among adolescents [14].

Studies on demographic differences have reported higher prevalence rates of IGD among males than among females [15–17]. Furthermore, IGD may be observed up to five times more often among males than among females in children and adolescents [18]. As adolescence is a period of transition, some of these minors might lack accurate knowledge of their own behaviour and sufficient self-control, and some might be particularly susceptible to factors associated with persistent on-/off-line gaming [19–21].

Symptoms of IGD and the underlying neurobiological mechanisms. Kuss et al. [22]. reported that compared with healthy controls, gaming addicted individuals have poorer response inhibition and emotion regulation, impaired prefrontal cortex function and cognitive control, poorer working memory and decision-making, and deficits in the neural reward system. Several studies of individuals with IGD have shown increased sensitivity to reward and decreased ability to control impulsivity in the face of reward stimuli [23, 24]. Additionally, IGD has also been linked to psychological detriments and negative consequences, such as negative and depressed moods [25], trait anxiety, social phobia, substance abuse and aggressive behaviours [26, 27], decreased conscientiousness [28], higher impulsivity [29] and lower self-esteem [30].

The socioenvironment is one of the crucial factors that plays a role in IGD [31]. Many studies have shown that adolescents with IGD are associated with parental and familial risk factors, including but not limited to insufficient parental care, oppressive and hostile parents, poor parent-child relations, and poor family cohesion and family violence [32, 33]. In addition, being bullied at school is more strongly associated with IGD [15].

The most consistent finding was that game design and video game type were related to IGD symptoms. Modern games provide communication platforms, instant feedback and rewards and are more likely to create an immersive experience for gamers, increasing gamer loyalty and engagement. A study of patients with GD who played ‘online’ games found that the two most common types of game played were massive multiplayer online role-playing games (MMORPGs) [7] and first-person shooters (FPSs) games [34].

Despite these contributions, there are still few studies focusing on the prevalence and possible risk factors of GD in adolescents based on the ICD-11 diagnostic guidelines. As GD is recognized as a behavioral addictions, it is necessary to identify risk factors that increase the likelihood of developing the disorder. To sum up, the current study, based on the ICD-11 GD diagnostic guidelines, aimed to investigate the prevalence and undergo a comprehensive synthesis to analyse risk factors such as sociodemographic predictors, psychological factors, parenting styles and negative life events, and game-related factors (e.g. gaming hours per week ) and GD among adolescents.

## Methods

### Participants and procedures

Using convenience sampling, 7901 middle school students were enrolled from twelve middle schools in Urumqi, Kashi and Bole City of Xinjiang Uygur Autonomous Region, China from 2020 to 2021. In each area, two junior high schools and two senior high schools were selected, and in each school, four classes were selected from each grade (grades 1–3 of junior high schools and grades 1–3 of senior high schools). The classes were randomly selected in each school. The inclusion criteria were as follows: being 12–18 years old, voluntary participating in the study; the subjects themselves and at least one of their parents/monitors completed the informed consent. The exclusion criteria for patients were: having mental illness or serious physical illness, having severe cognitive impairment, or any inability to fill out questionnaires.

Ultimately, a valid sample of 7790 students aged between 12 and 18 years (mean = 14.99; SD = 1.65; boy = 52.0%) participated in the current study, for an effective rate of 98.6%.

## Measures

### Sociodemographics and gaming-related behaviours

Sociodemographic data included participants’ age, gender, family structure (having siblings or being an only child), who the student lived with, and socioeconomic status (i.e., the highest level of education of family members, occupational and income strata of the family). Participants subjectively scored the occupational and income strata of the family members living with them from 1 (lowest) to 10 (highest). The detailed information on the occupational and income strata is presented in Supplementary Tables 1 and 2. Gaming-related behaviours included the types of games most often played and the game hours per week spent playing on primarily online, stand-alone and/or video games.

### Assessment of gaming disorder symptoms

The Gaming Disorder Symptom Questionnaire-21 (GDSQ-21) was used to assess GD [35], which consists of 21 items with three subscales, impaired control, increasing priority, and continued use despite the occurrence of negative consequences. This instrument is used to assess the severity of GD symptoms by examining both online and/or offline gaming activities occurring over a 12-month period. Participants indicated how much they agreed with the statements on a 5-point Likert scale: 0 (“hardly ever”), 1 (“less than once a month”), 2 (“once a month”), 3 (“once a week”), and 4 (“almost every day”). Its three-dimensional structure corresponds to the new ICD-11 diagnostic concept of GD. It has good validity and internal consistency, with a Cronbach’s alpha coefficient of 0.964, and plays an important role in the investigation of GD. The scoring is the same as that of the original version of the GDSQ-21: with the cutoff of ≥ 14 for impaired control dimension, ≥ 11 for increasing priority, ≥ 4 for continued, and ≥ 62 for the whole scale, met each dimension and total score can effectively screen for GD.

### Psychological status

To measure participants’ sensitivity to punishment and rewards, the Chinese version of the Behavioral Inhibition System and Behavioral Activation System (BIS/BAS) scale was used [36], it has 20 items to which participants respond on a 4-point Likert scale from 1(“totally agree”) to 4(“totally disagree”). The BIS/BAS consists of 20-item self-rating questionnaire with good psychometric properties. The questionnaire comprises 13 BAS items and 7 BIS items. The 3 subscales of the BAS assess responsiveness to reward, drive towards appetitive goals, and fun seeking. In the present study, the Cronbach’s alpha coefficient of the scale was 0.891.

### Emotion regulation

To assess an individual’s typical level of emotion dysregulation, the Chinese version of the Emotion Regulation Questionnaire (ERQ) scale was used [37]. The ERQ is a 10-item self-rating questionnaire of how frequently people use two emotion regulation strategies: cognitive reappraisal (6 items), an antecedent-focused strategy, and expressive suppression (4 items), a response-focused strategy. The items are rated on a 7-point Likert scale ranging from 1 (“strongly disagree”) to 7 (“strongly agree”). In this study, the Cronbach’s alpha coefficient of the scale was 0.967, which reflects good reliability.

### Family functioning

Parenting styles and behaviours were assessed by the Chinese version of the Short-form Egna Minnenav Barndoms Uppfostran for Chinese (s-EMBU-C) [38], which has 42 items, divided into paternal and maternal versions. Each version of the scale contains three subscales, namely, refusal, emotional warmth, and overprotection. All items are rated on a 4-point Likert scale, with each subscale being scored as follows: 1 (“never”), 2 (“occasionally”), 3 (“often”), and 4 (“always”). In the current study, the Cronbach’s alpha coefficient of the scale was 0.884.

### Negative life events

Negative life events were measured by the Adolescent Self-Rating Life Events Checklist (ASLEC) [39], which evaluates the impact of negative life events experienced within the past 12 months. The ASLEC consists of 26 items, including 6 subscales: interpersonal relationships, study pressure, being punished, bereavement, health adjustment and others. Each item is scored on a 5-point Likert scale ranging from 1 (“not at all”) to 5 (“extremely severe”). In our study, the Cronbach’s alpha coefficient of internal consistency was 0.938.

### Statistical analyses

First, based on the ICD-11 diagnostic guidelines, the GDSQ-21 cut-off point was used to calculate the prevalence of GD. Besides estimating prevalence, Univariate analyses were conducted: independent samples *t* tests and *chi-square* tests were used to further compare the sociodemographic variables, psychological factors, parenting styles and negative life events, and game-related factors (e.g., hours spent on game design and types of video games) of the two groups.

Second, binary logistic regressions analyses were carried out with the GDSQ-21 score as the dependent variable and sociodemographic variables, psychological factors, parenting styles and negative life events as independent variables. These variables are summarized in Supplementary Table 3.

Before conducting the regression analysis, to make the regression model more reasonable, it is necessary to first conduct univariate analysis to determine which variables are related to GD. To avoid omitting variables that may have an effect, variables with a statistically significant difference *p* < 0.2 should be considered for inclusion in the regression equation as potential predictors.

In addition, in terms of gaming behaviour characteristics, there were differences between GD and Non-GD. Theoretically, these characteristics could be the result of GD, the cause of GD, or the accompanying manifestations of the disorder. Thus, this study does not consider these gaming behaviour characteristics causal variables for the occurrence of GD, but there are differences between GD and Non-GD in these variables. Therefore, univariate analysis of gaming behaviour characteristics was also conducted.

All the statistical analyses were carried out with SPSS software (version 25.0).

## Results

### Prevalence of GD and sociodemographic characteristics among participants

We identified that the 12-month prevalence was 2.27% (*n* = 177) adolescents with GD and 97.73% (*n* = 7613) adolescents with Non-GD. All participants were divided into GD (boys/girls = 145/32, mean age: 15.62 ± 1.69) and Non-GD (boys/girls = 3597/4016, mean age: 14.98 ± 1.64). The detailed sociodemographic characteristics of the GD and the Non-GD are presented in Table 1. Compared to Non-GD, in the GD there were more boys (*χ²* = 83.31, *p* < 0.001), they were a little bit older (i.e., a higher proportion of senior grades), and more likely to be only children (*χ²* = 2.01, *p* < 0.01), who the student lives with (*χ²* = 16.50, *p* < 0.05). Those in the GD were in lower occupational strata by their family (*t* = 1.51, *p* < 0.05) and income strata by their family (*t* = 1.72, *p* < 0.2).

**Table 1:** Comparison of demographic characteristics between Non-GD and GD

Variable	No GD	GD	t/χ²	*p* Value
Age in years; mean (SD)	14.98 (1.64)	15.62 (1.69)	-4.97	< 0.001^***^
Gender (boys, n, %)	3597 (47.2)	145 (81.9)	83.31	< 0.001^***^
Family structure (n, %)			2.01	0.009^**^
Being an only child	3719 (48.9)	96 (54.2)		
Having siblings	3894 (51.1)	81 (45.8)		
Who the student lives with (n, %)			16.50	0.011^*^
Parents	5449 (71.6)	120 (67.8)		
Father only	168 (2.2)	4 (2.3)		
Mother only	799 (10.5)	24 (13.6)		
Grandparents only	129 (1.7)	3 (1.7)		
Parents and grandparents	476 (6.3)	433 (2.3)		
Living in the school dormitory	433 (5.7)	20 (11.3)		
Other	159 (2.1)	2 (1.1)		
The highest level of education of family members (n, %)			3.79	0.435
Primary school or below	260 (3.4)	3 (1.7)		
Junior high school	1582 (20.8)	34 (19.2)		
Senior high school or vocational high school	1773 (23.3)	36 (20.3)		
College or junior college	3570 (46.9)	94 (53.1)		
Master or above	428 (5.6)	10 (5.6)		
Occupational strata of the family^a^ (n, %)	5.30 (1.87)	5.09 (2.07)	1.51	0.007^**^
Family income strata of the family^a^ (n, %)	5.11 (1.81)	4.88 (1.89)	1.72	0.080

^***^p value < 0.001, ^**^p value < 0.01, ^*^p value < 0.05. ^a^: Family occupational strata (1~10): 1 indicates the lowest, and 10 indicates the highest; Family income strata  (1~10): 1 indicates the lowest, and 10 indicates the highest; *GD* Gaming disorder

### Gaming-related behaviours

Gaming-related behaviours for the whole sample, the GD and the Non-GD are presented in Table 2. Compared to the Non-GD, the GD spent significantly more hours on online or playing on-/offline games. Moreover, GD prefer to choose specific game genres.

**Table 2:** Comparison of game-related behaviours characteristics between Non-GD and GD

Variable	No GD	GD	t/χ²	*p* Value
Game hours per week online (in hours) (n, %)			625.18	< 0.001^***^
Less than 2 h	5091 (66.9)	15 (8.5)		
Between 2 and 4 h	1081 (14.2)	21 (11.9)		
Between 4 and 8 h	730 (9.6)	40 (22.6)		
Between 8 and 16 h	347 (4.6)	24 (13.6)		
Between 16 and 32 h	171 (2.2)	33 (18.6)		
Between 32 and 64 h	93 (1.2)	18 (10.2)		
Between 64 and 128 h	58 (0.8)	12 (6.8)		
More than 128 h per week	42 (0.6)	14 (7.9)		
Weekly stand-alone games (n, %)			1175.435	< 0.001^***^
Less than 2 h	6283 (82.5)	36 (20.30)		
Between 2 and 4 h	659 (8.7)	11 (6.2)		
Between 4 and 8 h	357 (4.7)	36 (20.3)		
Between 8 and 16 h	172 (2.3)	25 (14.1)		
Between 16 and 32 h	70 (0.9)	29 (16.4)		
Between 32 and 64 h	25 (0.3)	16 (9.0)		
Between 64 and 128 h	18 (0.2)	13 (7.3)		
More than 128 h per week	29 (0.4)	11 (6.2)		
Weekly video games (n, %)			43.087	< 0.001^***^
Less than 2 h	6380 (83.8)	34 (19.2)		
Between 2 and 4 h	621 (8.2)	16 (9.0)		
Between 4 and 8 h	314 (4.1)	42 (23.7)		
Between 8 and 16 h	141 (1.9)	18 (10.2)		
Between 16 and 32 h	83 (1.1)	27 (15.3)		
Between 32 and 64 h	35 (0.5)	10 (5.6)		
between 64 and 128 h	17 (0.2)	17 (9.6)		
More than 128 h per week	22 (0.3)	13 (7.3)		
Types of games played most often ( n, %)			43.087	< 0.001^***^
Multiplayer online battle arenas ( MOBAs)	2166 (39.1)	100 (61.0)		
First-person shooter (FPS)	771 (13.9)	20 (12.2)		
Building and management games (My World)	498 (9.0)	11 (6.7)		
Massively multiplayer online role-playing. games (MMORPG)	357 (6.4)	9 (5.5)		
Sports Games (FIFA)	345 (6.2)	5 (3.0)		
Real-time strategy games (RTS)	84 (1.5)	5 (3.0)		
Action games (ACT)	103 (1.9)	3 (1.8)		
Card games	151 (2.7)	2 (1.2)		
Other types	1067 (19.3)	9 (5.5)		

^***^p value < 0.001

### Univariate analysis of psychological factors

For reward processing, emotional characteristics, regulatory strategies, parenting styles and behaviours, and impact of negative life events (i.e., BIS/BAS, ERQ, s-EMBU-C, ASLEC questionnaires), *t* tests were used to determine the differences between the GD and the Non-GD, as shown in Table 3. The results showed that the GD and the Non-GD were significantly different in terms of BIS- behavioral inhibition (*t* = -1.933, *p* < 0.02); BAS- reward responsiveness (*t* = -4.659, *p* < 0.001) and drive (*t* = -3.451, *p* < 0.001); ERQ-expressive suppression (*t* = 0.480, *p* < 0.02); s-EMBU-C-paternal refusal (*t* = -6.745, *p* < 0.001), emotional warmth (*t* = 2.996, *p* < 0.01), overprotection (*t* = -5.213, *p* < 0.001), maternal refusal (*t* = -7.657, *p* < 0.001), emotional warmth (*t* = 2.936, *p* < 0.01), and overprotection (*t* = -5.419, *p* < 0.001); and ASLEC-interpersonal relationships (*t* = -7.756, *p* < 0.001), study pressure (*t* = -6.280, *p* < 0.001), being punished (*t* = -7.022, *p* < 0.001), bereavement (*t* = -3.458, *p* < 0.01), and health adjustment (*t* = -5.967, *p* < 0.001). No significant differences were detected between the GD and the Non-GD in terms of BAS-fun seeking or ERQ-cognitive reappraisal.

**Table 3:** Univariate analysis of psychological factors, parenting styles and negative life events between Non-GD and GD

Scales	Dimension	No GD	GD		
		Mean (SD)	Mean (SD)	t	*p* Value
BIS/BAS	BIS	15.22 (3.77)	14.63 (4.04)	-1.933	0.053
	BAS	23.92 (8.12)	25.84 (9.47)	-3.092	0.002^**^
	BAS- reward responsiveness	6.20 (2.44)	7.31 (3.14)	-4.659	< 0.001^***^
	BAS-drive	7.72 (2.96)	8.54 (3.11)	-3.451	< 0.001^***^
	BAS-fun seeking	10.0 (3.59)	9.99 (3.81)	0.031	0.975
ERQ	Cognitive reappraisal	26.92 (9.70)	26.62 (8.26)	0.412	0.632
	Expressive suppression	16.70 (6.39)	17.64 (5.94)	0.480	0.052
s-EMBU-c	Paternal refusal	8.74 (3.31)	10.79 (3.99)	-6.745	< 0.001^***^
	Paternal emotional warmth	19.79 (5.52)	18.54 (5.04)	2.996	0.003^**^
	Paternal overprotection	15.84 (4.06)	17.45 (3.85)	-5.213	< 0.001^***^
	Maternal refusal	8.59 (3.78)	10.92 (4.02)	-7.657	< 0.001^***^
	Maternal emotional warmth	21.49 (6.29)	20.09 (5.16)	2.936	0.003^**^
	Maternal overprotection	17.15 (4.58)	19.04 (4.09)	-5.419	< 0.001^***^
ASLEC	Interpersonal relationships	10.65 (4.31)	13.20 (4.67)	-7.756	< 0.001^***^
	Study pressure	10.58 (3.79)	12.40 (4.14)	-6.280	< 0.001^***^
	Being punished	10.59 (4.43)	13.93 (6.28)	-7.022	< 0.001^***^
	Bereavement	4.65 (2.34)	5.42 (2.92)	-3.458	0.001^**^
	Health adjustment	4.72 (2.03)	5.97 (2.77)	-5.967	< 0.001^***^

^***^p value < 0.001, ^**^p value < 0.01, ^*^p value < 0.05. *GD* Gaming Disorder, *BIS/BAS* Behavioral Inhibition System and Behavioral Activation System Scale, *ERQ* Emotion Regulation Questionnaire, *s-EMBU-c* Short-form Egna Minnenav Barndoms Uppfostran for Chinese, *ASLEC* Adolescent Self-Rating Life Events Checklist

### Logistic regression analysis

The results of the binary logistic regression analysis are shown in Table 4. Variables that were significantly different in the univariate analysis were included in the regression model as independent variables. These variables consisted of age; gender; being an only child; who the student lived with; family occupational strata, family income strata; BIS- behavioral inhibition; BAS-reward responsiveness and drive; ERQ-expressive suppression; s-EMBU-C-paternal and maternal of refusal, emotional warmth, and overprotection; and ASLEC-interpersonal relationships, study pressure, being punished, bereavement, and health adjustment.

**Table 4:** Binary logistic regression analysis of gaming disorder(GD)

Variable	Β	S.E	Wald	OR (95% CI)	*p* Value
Age	0.232	0.052	19.799	1.261 (1.139–1.397)	< 0.001^***^
Gender (vs. girl)	-1.557	0.205	57.515	0.211 (0.141–0.315)	< 0.001^***^
Being an only child (vs. having siblings)	-0.242	0.165	2.160	0.785 (0.569–1.084)	0.142
Who the student live with			12.248		0.057
Parents	0.285	0.730	0.152	1.329 (0.318–5.554)	0.696
Father only	0.131	0.895	0.021	1.140 (0.197–6.588)	0.884
Mother only	0.540	0.755	0.512	1.716 (0.391–7.535)	0.474
Grandparents only	0.426	0.937	0.207	1.532 (0.244–9.612)	0.649
Parents and grandparents	-0.641	0.883	0.528	0.527 (0.093–2.971)	0.468
Own Residence	0.983	0.759	1.678	2.674 (0.604–11.838)	0.195
Family occupational strata	0.039	0.063	0.380	1.039 (0.919–1.175)	0.537
Family income strata	-0.076	0.065	1.356	0.927 (0.815–1.053)	0.244
BIS-behavioral inhibition	-0.028	0.026	1.090	0.973 (0.924–1.024)	0.296
BAS- reward responsiveness	0.094	0.046	4.180	1.099 (1.004–1.202)	0.041^*^
BAS-drive	0.024	0.039	0.366	1.024 (0.948–1.106)	0.545
ERQ- expressive suppression	0.014	0.014	1.042	1.014 (0.987–1.042)	0.307
s-EMBU-C-paternal refusal	0.021	0.031	0.442	1.021 (0.960–1.085)	0.506
s-EMBU-C-paternal emotional warmth	-0.002	0.019	0.008	0.998 (0.963–1.035)	0.927
s-EMBU-C-paternal overprotection	-0.004	0.028	0.023	0.996 (0.942–1.052)	0.878
s-EMBU-C-maternal refusal	0.059	0.027	5.020	1.061 (1.007–1.118)	0.025^*^
s-EMBU-C-maternal emotional warmth	-0.021	0.017	1.484	0.979 (0.947–1.013)	0.223
s-EMBU-C-maternal overprotection	0.010	0.024	0.177	1.010 (0.963–1.059)	0.674
ASLEC-interpersonal relationships	0.067	0.028	5.599	1.070 (1.012–1.131)	0.018*
ASLEC-study pressure	-0.029	0.035	0.688	0.971 (0.906–1.041)	0.407
ASLEC-being punished	0.070	0.027	6.653	1.073 (1.017–1.132)	0.010^*^
ASLEC-bereavement	-0.151	0.046	10.517	0.860 (0.785–0.942)	0.001^**^
ASLEC-health adjustment	0.063	0.049	1.657	1.065 (0.968–1.171)	0.198
Constant	-6.995	1.344	27.105	0.001	< 0.001^***^

^***^p value < 0.001, ^**^p value < 0.01, ^*^p value < 0.05. *GD* Gaming Disorder, *BIS/BAS* Behavioral Inhibition System and Behavioral Activation System Scale, *ERQ* Emotion Regulation Questionnaire, *s-EMBU-c* Short-form Egna Minnenav Barndoms Uppfostran for Chinese, *ASLEC* Adolescent Self-Rating Life Events Checklist, *OR* Odds ratio

Regarding sociodemographic characteristics, age (odds ratio: 1.261) and gender (odds ratio: 0.211) were significantly associated with GD. The BAS-reward score (odds ratio:1.099) was a significant psychological predictor of GD. s-EMBU-C-maternal refusal (odds ratio: 1.061) was a significant family-environment predictor of GD. ASLEC-interpersonal relationships (odds ratio: 1.070), being punished (odds ratio: 1.073) and bereavement (odds ratio: 0.860) were significant socioenvironmental predictors of GD. Specifically, boys were 0.211 times more likely to have GD than girls. With a one-year increase in age, participants were 1.261 times more likely to be classified as having a GD (participants were between the ages of 12 and 18). With a one-unit score higher for the s-EMBU-C-maternal refusal, ASLEC- interpersonal relationships, being punished and bereavement factors, the probability of GD increased by 1.061, 1.070, 1.073 and 0.86 times, respectively.

## Discussion

This study aimed to investigate the prevalence of GD and analyze its possible risk factors of IGD among adolescents attending school in China based on the diagnostic guidelines the ICD-11 proposes. Three major findings emerged from this investigation: (1) Compared to the Non-GD, GD were a little bit older and more boys. (2) The hours spent on games playing are positively associated with GD. Moreover, GD preferred to choose specific game genres. (3) Some factors (psychological, parenting styles and negative life events) are associated with GD.

### Prevalence of GD

Our results reported the 12-month prevalence among adolescents of China. A recent study showed 2.2% of the 28 representative sample studies prevalence estimates, which is nearly comparable to our findings [40]. The increasing popularity of the Internet in China in recent years may contribute to the growing size of underage Internet users. The number of juvenile internet users reached 193 million in 2022 [41]. The proportion of underage Internet users who play computer games has also increased. Excessive gaming behavior and poor usage habits can be very engaging, time consuming and easily addictive to some gaming users. As young people are mentally and physically immature, their physical and mental health is highly susceptible to environmental and behavioral influences and they are more likely to develop mental health problems directly related to their gaming behavior.

### Sociodemographic characteristics of participants with GD

In terms of age, a greater proportion of patients in the GD were in the upper grades than those in the Non-GD. Previous studies have also suggested that age has an inverted U-shaped relationship with online GD [42, 43] and is correlated with the amount of hours spent playing games [44, 45]. Adolescents in higher grades were more likely to be gaming addicted [46].

Gender as a risk factor for GD. In this study, there were more boys with GD than girls. This finding is consistent with previous research showing that GD was more likely to occur in the male users [47–50]. This may be because male are more likely to choose competitive [51] and adversarial activities [52] for entertainment, and a significant proportion of all types of games have these characteristics. Compared to male gamers, female gamers engage in gaming for entertainment and socializing rather than to experience combat or to alleviate negative emotions [53–56], and they also spend less hours online and looking at the screen while playing [57]. In addition, behavioral addictions such as polysubstance dependence and gambling are generally more prevalent in males than females [58, 59]. This suggested that gender, addiction susceptibility and other factors may contribute to the greater susceptibility of males to addiction, which may similarly contribute to the higher prevalence of males than females with GD. In addition, brain imaging studies have shown that the males and females respond differently to gaming cues [60], with more activation in areas such as the striatum and orbitofrontal cortex in males, which may be the brain mechanism for the aforementioned gender differences [61].

The greater proportion of only children among those with GD (results of univariate analysis) is consistent with previous research findings that adolescents’ peer interactions are both necessary for their social needs and have recreational properties that allow them to enrich their leisure time [62]. The peer interactions of only children may be more limited to classmates and friends, and they may be more likely to find ways to interact with peers through the virtual world of online games when social needs are relatively inadequately met.

In terms of the level of education, occupational strata and income strata of the family members living with participants. Previous studies have suggested that the educational level of the family may be associated with the onset of GD [63], but this study failed to find a difference in this variable between GD and Non-GD. One of the reasons this study failed to find an association between parental education level and GD in adolescents may be that the popularity of smartphones and the mobile Internet in China in recent years has made people’s information sources extremely rich and significantly decreased the threshold of information access [64], allowing parents with different levels of education to learn about the dangers of GD and preventive measures, thus responding to adolescent gaming. This has led to a homogenization of the response to adolescent gaming problems, making the effect of education level of the family members less significant. On the other hand, this study revealed that family members with GD had lower occupational and income strata than Non-GD. Given that occupational and income are closely related and that higher occupational and income strata are more likely to help families provide good material and spiritual life for their adolescents, this study hypothesizes that gaming is a relatively accessible and inexpensive entertainment for adolescents with relatively low material and mental life, making them more likely to use gaming and more likely to develop GD.

### Game hours and game genre preference of GD

This study found that people with GD spent more hours and money on gaming-related behaviours than Non-GD, which is consistent with previous findings [65]. In addition, previous research has shown that males spend twice as more game hours as females playing video games each week and on weekends [66], an easily understandable characteristic that is more likely to promote GD as game immersion and game hours per week increase. Importantly, gamers with GD are also more likely to engage in in-game payment behaviour. In recent years, the gaming industry has proliferated the variety of their revenue streams (such as increasing membership fees and selling virtual props and cosmetic skins) [67]. In addition, online gaming is more addictive than offline gaming, possibly because of its inherent social reinforcement [68].

In terms of game genre preference, this study revealed that people with GD prefer specific game genres, considering that they may be designed to capture the attention of gamers and promote their addiction to endless gaming experiences, such as multiplayer online battle arenas (MOBAs) games, which provide a social environment built entirely on gaming screens. The short duration of these games, their ability to provide rapid positive feedback and their suitability for use on mobile phones may be important reasons games in this genre were widely played by those with GD. Future research should focus on the regulation of gaming products and genres to effectively prevent and control GD in the future.

### Psychological factors, parenting styles and negative life events associated with GD

Among the factors influencing GD, this study focused on psychological factors in terms of reward processing and emotional characteristics. Univariate analysis results showed that compared to Non-GD, GD have lower behavioral inhibition, having greater reward reactivity and drive, using more expressive inhibition in emotion regulation. The logistic regression revealed that the BIS inhibition dimension and ERQ did not predict GD among adolescents, whereas the reward response on the BAS activation dimension was predictive of GD. This suggesting that adolescents primarily seek pleasure and rewards when playing games and that many online games themselves allow players to earn points, level up, and obtain rewards that tend to induce positive pleasure and reward effects.

The parenting styles of paternal and maternal have an important influence on the development of GD among adolescents. Univariate analysis revealed that GD have more experiencing rejection parenting styles, having lower emotional warmth, and experiencing greater overprotection. According to the logistic regression model, maternal rejection was the only significant family correlate. The more the mother rejected and denied the child, used inappropriate parenting styles and punished the child too harshly, the more likely the child was to develop GD. In general, family parenting is a combination of both parents’ parenting attitudes and behaviours, but children general have the most contact with their mothers and develop more attachment to them, so the mother’s feedback and evaluation of the child is particularly important, especially during the adolescent years when self-perception is still unclear. If the mother has a clear tendency to reject the child and has disrespectful thoughts towards the child, this may have a greater negative impact on the child, which may increase more game hours the adolescent spends gaming.

Negative life events also play an important role in the development of gaming problems in adolescents. Univariate analyses revealed that GD have more experiencing more negative life events. The interpersonal relationships, being punished and bereavement factors were significant negative life events in the regression model. When adolescents felt stress or negative emotions in their interpersonal relationships and being punished, some of them adolescents began to use gaming as a way of relieving dissatisfaction and escaping from real-life problems. When experiencing life events such as the loss of family, friends and job, individuals may become consumed with grief and temporarily turn away from games. Later, when the individual is unable to avoid the event or solve the problem in the usual way, the individual may use game to escape from the sadness.

Mannikko et al [69]. suggested that excessive gameplay can harm adolescents on their psychological, social, and physical health. The results of our study showed that GD have higher reward responsiveness, more maternal rejecting and more occurrence of negative life events (e.g., interpersonal relationships, being punished and bereavement factors). In addition, many other possible risk factors for GD also needs to be further explored. The possible risk factors that have been identified carry clinical importance as they provide a more holistic approach towards adolescent in the prevention of GD.

### Limitations of study

As with all research, this study has some limitations and strengths. First, we used a convenience sampling method to conduct this research in twelve schools in the Xinjiang Uygur Autonomous Region, China, so the results should be validated in other regions of China in the future. Second, all of the assessments were self-reported and might have suffered from social desirability bias. Third, this study was that the GD was evaluated only the GDSQ-21, and no evaluation was performed according to psychiatric clinical diagnostic interviews. Therefore, the accuracy, specificity, and sensitivity could not be determined. Future research in the field should compare clinically diagnosed samples using actual GDSQ-21 test scores. Fourth, this study was cross sectional study, which prevents drawing conclusions about the causal relationships between variables. Therefore, further research is needed to explore these longitudinal relationships.

## Conclusion

In conclusion, the results of our study showed the prevalence of GD, and possible risk factors, which the reward responsiveness, maternal rejecting and occurrence of negative life events associated with GD. This study showed a positive association between gaming hours and GD, and GD preferred to choose a specific game genre. Furthermore, we found that gender and age were significant predictors of gaming harm with a little bit older males (i.e., a higher proportion of senior grades) being more susceptible. This study may provide some suggestion for real life. In prevention and intervention, prevention efforts should aim at identifying possible risk factors.

### Electronic supplementary material

Below is the link to the electronic supplementary material.

Supplementary Material 1

## Data Availability

The datasets used and/or analyzed during the present study are available from the corresponding author upon reasonable request.

## Abbreviations

DSM-5: Diagnostic and Statistical Manual of Mental Disorders
IGD: Internet gaming disorder
ICD-11: International Classification of Diseases 11th Revision
GD: Gaming disorder
GDSQ-21: Gaming Disorder Symptom Questionnaire-21
BIS/BAS: Behavioral Inhibition System and Behavioral Activation System Scale
ERQ: Emotion Regulation Questionnaire
s-EMBU-C: Short-form Egna Minnenav Barndoms Uppfostran for Chinese
ASLEC: Adolescent Self-Rating Life Events Checklist
OR: Odds ratio
