# Supplementary material for: The prevalence and possible risk factors of gaming disorder among adolescents in China
Source: BMC Psychiatry. 2024 May 21;24:381. doi: 10.1186/s12888-024-05826-9 (PMC11110186; doi:10.1186/s12888-024-05826-9)
Supplement: Supplementary file 1 — Supplementary Material 1 [file 12888_2024_5826_MOESM1_ESM.docx]

**Supplementary Table S1** Family social stratum

| Stratum | Occupational stratum of the family |
| --- | --- |
| 1 | state and social administrators |
| 2 | managers |
| 3 | private entrepreneurs |
| 4 | professionals and technicians |
| 5 | clerks |
| 6 | self-employed |
| 7 | employees in commercial services |
| 8 | industrial workers |
| 9 | agricultural labourers |
| 10 | urban and rural unemployed and semiunemployed persons |

Family occupational stratum: 1 indicates the lowest, and 10 indicates the highest

**Supplementary Table S2** Family income stratum

| Stratum | Family income stratum | Annual household income (yuan) |
| --- | --- | --- |
| 1 | extremely poor | $10,000 ~ $20,000 |
| 2 | poor | low-income30,000 ~ $60,000 |
| 3 | low-income | $60,000 ~ $150,000 |
| 4 | well-off | $150,000 ~ $300,000 |
| 5 | middle class | $300,000 ~ $500,000 |
| 6 | high income | $500,000 ~ $1,000,000 |
| 7 | slightly wealthy | $1,000,000 ~ $8,000,000 |
| 8 | moderately wealthy | $8,000,000 ~ $150,000,000 |
| 9 | luxuriously wealthy | $150,000,000 ~ $800,000,000 |
| 10 | extremely wealthy | ≥$800,000,000 |

Family income stratum: 1 indicates the lowest, and 10 indicates the highest

**Supplementary Table S3** Description of game-related behaviours characteristics, psychological factors and socioenvironmental influences.

| **Variales** | | **# of Items** |
| --- | --- | --- |
| Demographic characteristics | Age, gender, family structure | 7 |
|  | Who the student lives with |  |
|  | The highest level of education of family members Occupational/family income stratum of the family |  |
| game-related behaviours | the types of games played most often, the weekly time spent playing primarily online, stand-alone and/or video games | 4 |
| Assessment of GD Symptoms | GDSQ-21 | 7 |
| psychological factors | BIS | 7 |
|  | BAS- reward responsiveness | 5 |
|  | BAS-drive towards appetitive goals | 4 |
|  | BAS-fun seeking | 4 |
|  | ERQ-Cognitive Reappraisal | 6 |
|  | ERQ- Expressive Suppression | 4 |
| socioenvironmental influences | s-EMBU-c-paternal refusal, emotional warmth, overprotection | 21 |
|  | s-EMBU-c- maternal refusal, emotional warmth, overprotection | 21 |
|  | ASLEC-interpersonal relationships | 5 |
|  | ASLEC- study pressure | 5 |
|  | ASLEC- being punished | 7 |
|  | ASLEC- bereavement | 3 |
|  | ASLEC- health adjustment | 4 |

*GD* Gaming Disorder; *GDSQ-21* Gaming Disorder Symptom Questionnaire-21; *BIS/BAS* Behavioral Inhibition System and Behavioral Activation System Scale; *ERQ* Emotion Regulation Questionnaire; *s-EMBU-c* Short-form Egna Minnenav Barndoms Uppfostran for Chinese; *ASLEC* Adolescent Self-Rating Life Events Checklist
